# Association of blood manganese and selenium levels with hepatic steatosis among adolescents: a nationwide cross-sectional analysis

**DOI:** 10.3389/fped.2025.1522219

**Published:** 2025-02-11

**Authors:** Bin You, Zhiyuan Chen

**Affiliations:** ^1^ Department of Pediatrics, The Wenzhou Third Clinical Institute Affiliated to Wenzhou Medical University, Wenzhou, Zhejiang, China; ^2^ Department of Pediatrics, Wenzhou People’s Hospital, Wenzhou, Zhejiang, China; ^3^ Department of Pediatrics, Wenzhou Maternal and Child Health Care Hospital, Wenzhou, Zhejiang, China; ^4^ Department of Pediatrics, The Third Affiliated Hospital of Shanghai University, Wenzhou, Zhejiang, China

**Keywords:** selenium, manganese, hepatic steatosis, adolescents, NHANES

## Abstract

**Objective:**

This study aimed to investigate the association between blood manganese and selenium levels and hepatic steatosis among adolescents, using data from the National Health and Nutrition Examination Survey (NHANES) 2017–2023.

**Methods:**

A cross-sectional analysis was conducted using data from 2,459 adolescents (aged 12–19 years) with complete data on liver ultrasound transient elastography, blood manganese, and selenium levels. Hepatic steatosis was defined as a controlled attenuation parameter (CAP) score of ≥248 dB/m, a measure of liver steatosis, which is a primary characteristic and a less severe stage of hepatic steatosis, assessed by vibration-controlled transient elastography (VCTE). Multivariate logistic regression models were used to assess the associations between blood manganese and selenium levels and hepatic steatosis, while restricted cubic splines (RCS) were employed to examine the dose-response relationships.

**Results:**

The mean age of the participants was 15.37 years, with 52.22% boy. Higher blood manganese and selenium levels were significantly associated with an increased prevalence of hepatic steatosis. In the fully adjusted model, adolescents in the highest quartile of blood manganese had more than twice the odds of hepatic steatosis compared to those in the lowest quartile (OR = 2.41, 95% CI: 1.55–3.75, *P* < 0.01). Similarly, the highest quartile of blood selenium was associated with a 57% increase in hepatic steatosis prevalence compared to the lowest quartile (OR = 1.57, 95% CI: 1.19–2.08, *P* < 0.01). RCS analysis confirmed a linear association between both blood manganese and selenium levels and hepatic steatosis prevalence. Subgroup analyses did not reveal statistically significant interactions by age, sex, or obesity status, although associations appeared stronger in younger adolescents.

**Conclusion:**

Elevated blood manganese and selenium levels are associated with a higher prevalence of hepatic steatosis in adolescents. These findings suggest a potential role of trace elements in the development of hepatic steatosis, highlighting the need for further research to better understand the underlying mechanisms involved in liver fat accumulation in this population.

## 1 Introduction

Hepatic steatosis has emerged as the most common chronic liver disease in children and adolescents, paralleling the global rise in obesity. This initial stage of hepatic steatosis is characterized by excessive fat accumulation in the liver. It is often asymptomatic and follows a generally benign and non-progressive course in many individuals (1). Hepatic steatosis is marked by inflammation and liver cell damage alongside fat accumulation (1). Over recent decades, there has been a significant increase in obesity rates among children and adolescents globally. In the United States, the prevalence of non-alcoholic fatty liver disease (NAFLD) among children has more than doubled over the past 20 years (2). Studies suggest that the prevalence of NAFLD in overweight children aged 2–19 years ranges from approximately 9%–37% (3). As children grow, their risk of developing hepatic steatosis becomes more pronounced (4). The hepatic steatosis is becoming more prevalent among children and adolescents, with an annual increase of 1.35% from 1990 to 2017 (5). In a recent study, histological examinations revealed that hepatic steatosis was present in 4.5% of the unexpected childhood deaths in New York City (6).

Due to its role as a primary site for heavy metal storage, the liver is highly susceptible to toxicity from environmental pollutants, especially as heavy metal pollution continues to rise. This is crucial in studying metabolic diseases like hepatic steatosis. The hepatotoxic effects of selenium overexposure have been recognized for nearly a century, with the earliest reports dating back to the 1930s (7). Adolescents are especially at risk due to physiological and developmental factors, which may compromise the liver's detoxification ability and elevate the risk of metabolic disorders like hepatic steatosis (8).

The relationship between manganese and hepatic steatosis is complex and controversial. Some studies reported higher manganese blood levels were associated with an increased risk of hepatic steatosis (9). Elevated manganese levels may exacerbate liver injury by promoting oxidative stress and impairing mitochondrial energy production, thereby increasing the risk of hepatic steatosis progression toward more severe liver conditions. However, other research suggests beneficial effects of higher manganese intake, which could possibly mitigate metabolic dysfunction-associated steatotic liver disease (MASLD) (10). Higher serum manganese levels have also been shown to exert a protective influence on males with hepatic steatosis in a cohort-based case-control study of Chinese adults (11). Moreover, findings from the Japan Collaborative Cohort Study suggest that higher dietary manganese intake could reduce the likelihood of liver cancer in males and lower cardiovascular disease mortality (12, 13). While the potential benefits of manganese are evident, more research is necessary to clarify how manganese specifically affects hepatic steatosis progression.

Selenium is a trace mineral vital for various physiological processes, required in only small amounts, and is a key component of selenoproteins that help maintain the balance of oxidation and reduction within cells (14). Although selenium concentrations have been extensively studied in the context of liver diseases, the specific relationship between selenium levels and hepatic steatosis remains ambiguous. Higher selenium levels may be associated with increased risk of liver conditions (15), while others indicate potential protective effects (16). Though selenium is essential for human health, excessive or prolonged exposure can lead to its accumulation in the body, potentially causing toxicity. The degree of selenium accumulation and toxicity largely depends on the chemical form of selenium, as certain forms, such as selenomethionine and selenocysteine (17, 18), are more readily absorbed and incorporated into proteins, whereas others, like selenite and selenate (19, 20), may be more toxic due to their higher bioavailability and reactivity. The duration and intensity of exposure also play a significant role in determining the level of toxicity.

Given the complexities surrounding the relationship between metals and hepatic steatosis, continued investigation is essential to unravel the nuances of their impact on liver health. Despite the growing body of literature on this topic, evidence from children and adolescents remains scarce, particularly regarding whether blood manganese and selenium concentrations are associated with hepatic steatosis in this age group. Adolescents represent a unique and vulnerable population, as metabolic processes and nutrient needs during this developmental period may influence the risk of liver diseases like hepatic steatosis. Our study aims to fill this gap by examining the relationships between serum manganese and selenium levels and the prevalence of hepatic steatosis in a nationally representative sample of adolescents using data from the National Health and Nutrition Examination Survey (NHANES), contributing to a better understanding of how these trace metals may impact liver health during a critical stage of life.

## 2 Methods

### 2.1 Study population

This cross-sectional study utilized data from the NHANES, a program conducted by the National Center for Health Statistics (NCHS) that provides nationally representative health and nutritional data of the U.S. population. NHANES employs a stratified, multistage probability sampling design to ensure representation of various demographic subgroups to reflect the civilian, non-institutionalized U.S. population. Detailed protocols for participant recruitment, data collection, and measurements are publicly available (21). All participants provided informed consent, and the study protocols were approved by the NCHS Research Ethics Review Board.

**Figure 1:**
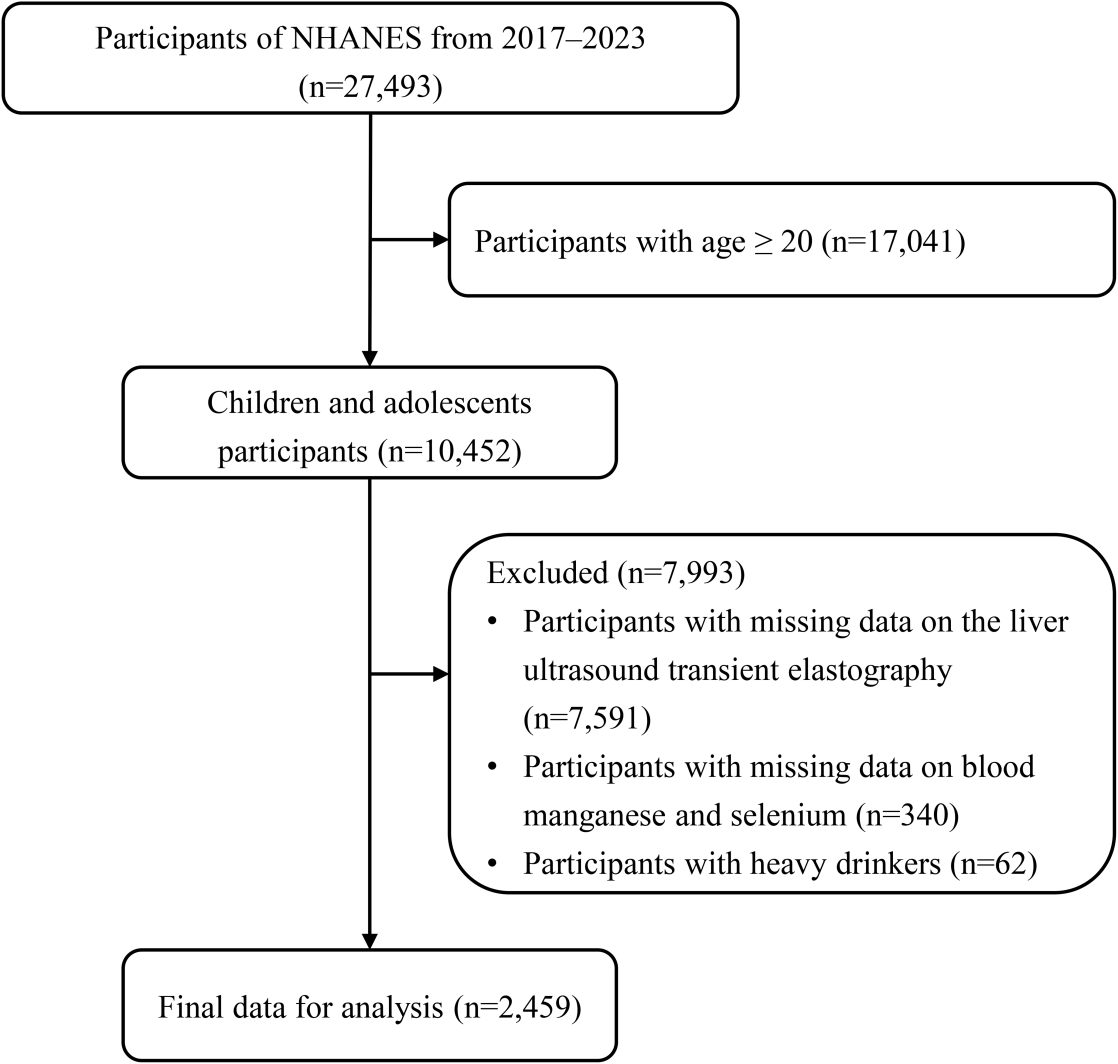
Flowchart of participants.

The study initially included 27,493 participants from the 2017 to 2023 NHANES cycles, consisting of 17,041 adults (≥20 years) and 10,452 children and adolescents. Since liver ultrasound transient elastography data were only available for adolescents and adults, 7,591 participants, including all younger children, were excluded due to missing liver elastography data. Further exclusions were made for missing blood manganese and selenium data (*n* = 340) and heavy alcohol consumption (*n* = 62). After these exclusions, a total of 2,459 adolescents remained in the final analysis (Figure 1).

### 2.2 Measurement of blood manganese and selenium

Whole blood biospecimens were collected during the NHANES examination, and the frozen samples were stored at −30°C before being transported for analysis at the National Center for Environmental Health, Centers for Disease Control and Prevention (CDC) in Atlanta, GA. Blood manganese and selenium levels were measured using inductively coupled plasma mass spectrometry (ICP-MS), a sensitive and reliable method for quantifying trace metals in biological specimens (22). Whole blood samples were prepared by a simple dilution process to ensure uniform distribution of cellular components, which is critical for accurately measuring metal concentrations. During this step, a small aliquot of blood was extracted from the well-mixed sample to reflect the average metal concentration across the entire specimen. The diluted blood samples were then introduced into the ICP-MS, where the sample was aerosolized and ionized in a high-temperature plasma. The ions generated from manganese and selenium were separated and detected based on their mass-to-charge ratios. To ensure accurate measurement and minimize interference from other ions, the mass spectrometer was operated in dynamic reaction cell (DRC) mode. Methane gas was used to remove potential interferences for selenium, while oxygen gas was employed to reduce interferences for manganese. Quality control procedures followed NHANES protocols, including the use of internal standards and regular calibration with known concentrations. All analyses met the quality assurance criteria established by the Centers for Disease Control and Prevention (CDC) to ensure precision and accuracy (23).

### 2.3 Definition of hepatic steatosis

In this study, hepatic steatosis was defined using vibration-controlled transient elastography (VCTE) with the FibroScan® model 502 V2 Touch. This non-invasive method quantifies liver fat via the controlled attenuation parameter (CAP), which ranges from 100 to 400 dB/m, with higher values indicating more fat (24, 25). Hepatic steatosis was defined as a CAP score of ≥248 dB/m, a threshold that balances sensitivity and specificity for detecting hepatic steatosis (26). While liver biopsy remains the diagnostic gold standard, VCTE is commonly used in epidemiological studies for its non-invasive nature and greater accuracy compared to standard ultrasound (27). Participants with excessive alcohol intake, which could affect liver fat and fibrosis, were excluded to ensure the study focused solely on metabolic-related fatty liver disease. This approach ensures accurate identification of hepatic steatosis in the study population.

### 2.4 Covariates

Several covariates were included to adjust for potential confounders in the association between blood manganese, selenium levels, and hepatic steatosis. Age was treated as a continuous variable, while sex was categorized as boys or girls. Race/ethnicity was divided into non-Hispanic White, non-Hispanic Black, and other races. Socioeconomic status was measured using the family poverty income ratio (PIR), which reflects the ratio of family income to the federal poverty level, adjusted for family size. PIR was categorized into three groups: ≤1.0 (at or below the poverty line), 1.1–3.0 (moderate income), and >3.0 (high income). Total cholesterol and high-density lipoprotein (HDL) cholesterol levels were included as continuous variables to assess lipid profiles. Body mass index (BMI) was treated as a continuous variable, and adolescent obesity was defined using CDC guidelines as a BMI at or above the 95th percentile for age and sex (28). Hypertension in adolescents was defined as either a reported diagnosis by a clinician, the use of antihypertensive medication, or a systolic blood pressure (BP) ≥130 mmHg and/or diastolic BP ≥ 80 mmHg, according to the 2017 American Academy of Pediatrics (AAP) guidelines (29). Diabetes was defined according to American Diabetes Association (ADA) criteria (30), which include fasting plasma glucose ≥126 mg/dl, 2-hour plasma glucose ≥200 mg/dl following an oral glucose tolerance test, or an HbA1c level ≥6.5%. Adolescents taking antidiabetic medications were also classified as having diabetes, regardless of blood glucose levels.

### 2.5 Statistical analysis

All statistical analyses were conducted using the R software (version 4.3.2). To ensure nationally representative estimates, we performed weighted analyses according to guidelines from the National Center for Health Statistics (NCHS), incorporating the MEC exam weights, strata, and primary sampling units (PSUs) using the “survey” package in R. Continuous variables with a normal distribution were compared using *t*-tests or one-way analysis of variance (ANOVA), while the Wilcoxon rank-sum test was applied for non-normally distributed variables. Categorical variables were analyzed using chi-square tests. The correlation between blood manganese and selenium levels and CAP scores was assessed using Spearman's rank correlation.

Multivariable linear regression was used to evaluate the relationship between blood manganese and selenium levels and CAP scores. Additionally, multivariate logistic regression was employed to explore associations between blood manganese and selenium levels and the prevalence of hepatic steatosis. Model 1 was adjusted for age (continuous), sex (boys or girls), and race (Mexican American, other Hispanic, non-Hispanic White, non-Hispanic Black, or other race); Model 2 was adjusted for Model 1 plus family poverty income ratio (≤1.0, 1.1–3.0, or >3.0), total cholesterol (continuous), high-density lipoprotein cholesterol (continuous), BMI (continuous), hypertension (no, or yes), and diabetes (no, or yes). Restricted cubic splines (RCS) with three knots at the 10th, 50th, and 90th percentiles were used to investigate the dose-response relationship between blood manganese and selenium levels and hepatic steatosis prevalence. Stratified analyses were conducted to examine how age, sex, race, family PIR, and obesity might influence these associations. A two-sided *P* value of less than 0.05 was considered statistically significant.

## 3 Results

### 3.1 Participant characteristics

The study included a total of 8,191 adolescents from the NHANES 2017–2023 cycles (Table 1). The mean age of participants was 15.37 years, with a balanced distribution of sex: 52.22% were male and 47.78% were female. The overall mean CAP score, reflecting liver fat content, was 220.05 dB/m. Significant differences were observed in blood manganese and selenium levels between adolescents with and without hepatic steatosis. Participants with hepatic steatosis had higher median blood manganese levels (10.89 μg/L) compared to those without hepatic steatosis (9.90 μg/L, *P* < 0.01). Similarly, blood selenium levels were higher in the hepatic steatosis group (180.30 μg/L) than in the participants without hepatic steatosis group (176.44 μg/L, *P* = 0.01). Other covariates showed notable group differences. Adolescents with hepatic steatosis had higher total cholesterol levels, lower HDL cholesterol levels, higher BMI, and a greater prevalence of hypertension and diabetes compared to those without hepatic steatosis. Additionally, race/ethnicity and family poverty income ratio (PIR) differed between the groups.

**Table 1:** Survey-weighted, sociodemographic and health status characteristics of adolescent participants in NHANES 2017–2023.

Characteristics	Total (*n* = 2,459)	Hepatic steatosis		*P* value
		No (*n* = 1,789)	Yes (*n* = 670)	
Age, years	15.37 ± 0.09	15.31 ± 0.11	15.52 ± 0.08	0.1
Sex, %				0.44
Girls	1,177 (47.78)	864 (48.37)	313 (46.15)	
Boys	1,282 (52.22)	925 (51.63)	357 (53.85)	
Race/ethnicity, %				<0.01
Mexican American	400 (14.62)	241 (11.52)	159 (23.11)	
Other Hispanic	295 (11.42)	214 (10.73)	81 (13.29)	
Non-Hispanic White	832 (47.73)	633 (50.86)	199 (39.14)	
Non-Hispanic Black	502 (12.52)	383 (12.94)	119 (11.38)	
Other race	430 (13.71)	318 (13.94)	112 (13.08)	
Family PIR, %				0.01
≤1.0	699 (21.93)	477 (20.23)	222 (26.59)	
1.1–3.0	1,012 (38.38)	726 (37.42)	286 (41.02)	
>3.0	748 (39.68)	586 (42.35)	162 (32.40)	
TC, mg/dl	154.88 ± 0.91	153.21 ± 1.05	159.44 ± 1.56	<0.01
HDL-C, mg/dl	51.85 ± 0.38)	53.51 ± 0.43	47.31 ± 0.48	<0.01
Body mass index, kg/m^2^	24.35 ± 0.20)	22.47 ± 0.21	29.51 ± 0.38	<0.01
Hypertension, %				
No	2,307 (94.67)	1,714 (96.45)	593 (89.79)	
Yes	152 (5.33)	75 (3.55)	77 (10.21)	
Diabetes, %				0.01
No	2,438 (99.38)	1,780 (99.63)	658 (98.68)	
Yes	21 (0.62)	9 (0.37)	12 (1.32)	
Blood manganese, μg/L	10.16 (8.29, 12.61)	9.90 (8.03, 12.19)	10.89 (8.97, 13.55)	<0.01
Blood selenium, μg/L	177.90 (165.30, 192.80)	176.44 (164.90, 191.95)	180.30 (165.90, 196.90)	0.01
CAP scores, dB/m	220.05 (1.23)	195.90 (1.04)	286.22 (1.74)	<0.01

PIR, poverty income ratio; TC, total cholesterol; HDL-C, high-density lipoprotein cholesterol; CAP, controlled attenuated parameter. Normally distributed continuous variables are described as means ± SEs, and continuous variables without a normal distribution are presented as medians (interquartile ranges). Categorical variables are presented as numbers (percentages). *N* reflects the study sample, whereas percentages reflect the survey-weighted data.

Survey-weighted characteristics of adolescent participants by quartiles of blood manganese (Supplementary Table S1) and selenium levels (Supplementary Table S2) were also analyzed. The results indicated that higher quartiles of both blood manganese and selenium were associated with increased CAP scores, suggesting a potential link between elevated levels of these trace elements and greater liver fat accumulation. Supplementary Figure S1 shows the Spearman correlation analysis between blood manganese, selenium levels, and CAP scores among adolescents. A weak but significant positive correlation was found between blood manganese and CAP (*r* = 0.09, *P* < 0.01), as well as between blood selenium and CAP (*r* = 0.05, *P* < 0.01).

### 3.2 Associations of blood manganese and selenium levels with hepatic steatosis

Supplementary Table S3 summarizes the associations between quartiles of blood manganese and selenium levels and CAP scores. In the fully adjusted model, higher quartiles of blood manganese were significantly associated with higher CAP scores, indicating greater liver fat accumulation. A similar trend was observed for blood selenium, with the highest quartile showing a significant increase in CAP scores compared to the reference group.

**Figure 2:**
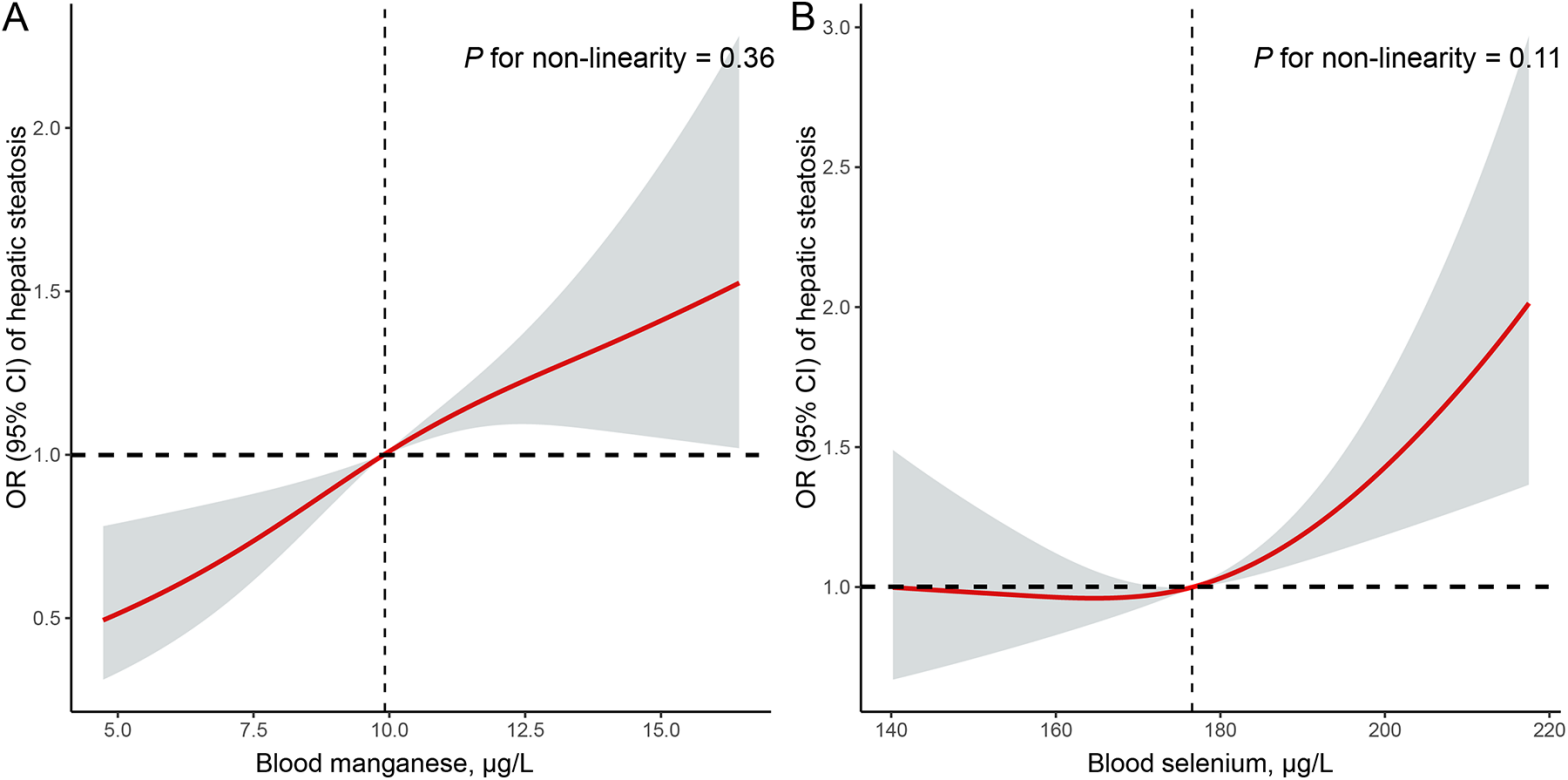
Restricted cubic spline (RCS) analysis with multivariate-adjusted associations of blood manganese **(A)** and selenium **(B)** levels and the prevalence of hepatic steatosis among adolescents in NHANES 2017–2023. RCS analysis was adjusted for age (continuous), sex (boys or girls), race (Mexican American, other Hispanic, non-Hispanic White, non-Hispanic Black, or other race), family poverty income ratio (≤1.0, 1.1 −3.0, or >3.0), total cholesterol (continuous), high-density lipoprotein cholesterol (continuous), BMI (continuous), hypertension (no, or yes), and diabetes (no, or yes).

Table 2 presents the associations between quartiles of blood manganese and selenium levels and the prevalence of hepatic steatosis. Higher quartiles of blood manganese were strongly associated with increased odds of hepatic steatosis. In the fully adjusted model, participants in the highest quartile of blood manganese had more than twice the odds of hepatic steatosis compared to those in the lowest quartile (OR = 2.41, 95% CI: 1.55–3.75, *P* < 0.01). Similarly, for blood selenium, the highest quartile was associated with significantly higher odds of hepatic steatosis (OR = 1.57, 95% CI: 1.19–2.08, *P* < 0.01) compared to the lowest quartile. Additionally, Figure 2 shows the RCS analysis of blood manganese and selenium levels in relation to hepatic steatosis prevalence among adolescents. As blood manganese levels increased, the odds of hepatic steatosis rose in a clear linear pattern (*P* for non-linearity = 0.32). Similarly, higher blood selenium levels were linked to an increased risk of hepatic steatosis, also following a linear trend (*P* for non-linearity = 0.09).

**Table 2:** Multiple logistic regression associations of quartiles of blood manganese and selenium levels with the prevalence of hepatic steatosis among adolescents in NHANES 2017–2023.

	Quartiles of blood manganese and selenium levels				*P*_trend_ *
	OR	OR (95% CI)	OR (95% CI)	OR (95% CI)	
Blood manganese					
Range	<8.29	8.29–10.16	10.17–12.61	>12.61	
Case (%)	122 (16.79)	160 (26.88)	179 (27.89)	209 (35.50)	
Crude	1 [Reference]	1.82 (1.37–2.42)	1.92 (1.47–2.50)	2.73 (1.80–4.13)	<0.01
Model 1	1 [Reference]	1.80 (1.34–2.41)	1.87 (1.44–2.44)	2.71 (1.83–4.01)	<0.01
Model 2	1 [Reference]	1.74 (1.23–2.46)	1.89 (1.33–2.67)	2.39 (1.54–3.71)	<0.01
Blood selenium					
Range	<165.30	165.30–177.90	177.91–192.80	>192.80	
Case (%)	161 (25.24)	135 (22.28)	169 (27.35)	205 (32.18)	
Crude	1 [Reference]	0.85 (0.64–1.13)	1.11 (0.77–1.61)	1.40 (1.09–1.80)	0.01
Model 1	1 [Reference]	0.85 (0.65–1.13)	1.09 (0.77–1.53)	1.40 (1.09–1.79)	0.01
Model 2	1 [Reference]	0.91 (0.62–1.33)	1.18 (0.76–1.82)	1.55 (1.15–2.08)	0.01

OR, odds ratio; CI, confidence interval; CAP, controlled attenuated parameter. Model 1 was adjusted for age (continuous), sex (boys or girls), and race (Mexican American, other Hispanic, non-Hispanic White, non-Hispanic Black, or other race); Model 2 was adjusted for Model 1 plus family poverty income ratio (≤1.0, 1.1–3.0, or >3.0), total cholesterol (continuous), high-density lipoprotein cholesterol (continuous), BMI (continuous), hypertension (no, or yes), and diabetes (no, or yes).

* *P* values for trend were calculated by treating the quartile groups as ordinal variables in the model.

### 3.3 Subgroup analysis

Subgroup analyses examined the associations between blood manganese and selenium levels and hepatic steatosis prevalence, focusing on age, sex, and obesity status (Tables 3, 4). For blood manganese, the association with hepatic steatosis appeared stronger among younger adolescents (aged 12–15 years) compared to older ones, though the interaction by age was not statistically significant (*P* for interaction = 0.28). While there were observed differences between boys and girls, the interaction by sex was also not significant (*P* for interaction = 0.61). The association between manganese levels and hepatic steatosis did not significantly vary by obesity status or other covariates. For blood selenium, a similar pattern was seen, with a stronger association in younger adolescents and minimal differences by sex and obesity, but none of these interactions reached statistical significance (*P* for interaction > 0.05). The effects of family income, cholesterol levels, and other covariates also showed no significant interactions with selenium levels.

**Table 3:** Stratified analyses of the associations between quartiles of blood manganese levels and the prevalence of hepatic steatosis among adolescents in NHANES 2017–2023.

Subgroups	*N*	Quartiles of blood manganese levels, μg/L				*P*_interaction_ *
		<8.29	8.29–10.16	10.17–12.61	>12.61	
Age, years						0.28
12–15	1,272	1 [Reference]	1.81 (1.10–3.00)	2.22 (1.11–4.47)	3.43 (1.49–7.86)	
16–19	1,187	1 [Reference]	1.61 (0.96–2.73)	1.54 (0.93–2.57)	1.76 (1.01–3.05)	
Sex, %						0.60
Girls	1,177	1 [Reference]	2.16 (1.28–3.64)	2.42 (1.49–3.95)	2.45 (1.41–4.25)	
Boys	1,282	1 [Reference]	1.42 (0.70–2.86)	1.45 (0.87–2.43)	2.22 (1.15–4.29)	
Race, %						0.25
Mexican American	400	1 [Reference]	2.34 (0.88–6.19)	3.12 (1.52–6.41)	3.61 (1.17–11.09)	
Other Hispanic	295	1 [Reference]	4.62 (1.07–19.99)	4.37 (1.07–17.90)	4.39 (0.91–21.10)	
Non-Hispanic White	832	1 [Reference]	1.77 (1.05–2.99)	1.20 (0.72–2.01)	3.06 (1.68–5.59)	
Non-Hispanic Black	502	1 [Reference]	1.48 (0.69–3.18)	3.36 (1.14–9.90)	2.94 (1.37–6.33)	
Other race	430	1 [Reference]	1.30 (0.33–5.09)	2.30 (0.68–7.77)	1.38 (0.58–3.30)	
Family PIR, %						0.69
≤1.0	699	1 [Reference]	1.39 (0.64–3.02)	2.01 (0.93–4.35)	2.46 (0.97–6.24)	
1.1–3.0	1,012	1 [Reference]	1.99 (1.17–3.38)	2.11 (1.11–4.01)	3.64 (2.04–6.51)	
>3.0	748	1 [Reference]	2.01 (1.05–3.87)	1.80 (0.70–4.61)	1.78 (0.73–4.36)	
Obesity, %						0.57
No	73.6	1 [Reference]	2.06 (1.08–3.94)	1.86 (0.84–4.12)	2.51 (1.34–4.72)	
Yes	26.4	1 [Reference]	1.32 (0.81–2.15)	1.88 (1.10–3.20)	2.28 (1.29–4.03)	

OR, odds ratio; CI, confidence interval. Analyses were adjusted for covariates age (continuous), sex (boys or girls), race (Mexican American, other Hispanic, non-Hispanic White, non-Hispanic Black, or other race), family poverty income ratio (≤1.0, 1.1–3.0, or >3.0), total cholesterol (continuous), high-density lipoprotein cholesterol (continuous), BMI (continuous), hypertension (no, or yes), and diabetes (no, or yes) when they were not the strata variables.

* *P* for interaction were derived using the likelihood ratio test by including interaction terms between the variables of interest in the model.

**Table 4:** Stratified analyses of the associations between quartiles of blood selenium levels and the prevalence of hepatic steatosis among adolescents in NHANES 2017–2023.

Subgroups	*N*	Quartiles of blood selenium levels, μg/L				*P*_interaction_ *
		<165.30	165.30–177.90	177.91–192.80	>192.80	
Age, years						0.611
12–15	1,272	1 [Reference]	1.13 (0.62–2.07)	1.22 (0.66–2.26)	1.76 (1.00–3.10)	
16–19	1,187	1 [Reference]	0.81 (0.53–1.23)	1.14 (0.69–1.89)	1.34 (0.90–2.01)	
Sex, %						0.27
Girls	1,177	1 [Reference]	0.90 (0.52–1.56)	0.96 (0.52–1.77)	1.71 (1.18–2.46)	
Boys	1,282	1 [Reference]	0.91 (0.59–1.42)	1.52 (0.91–2.51)	1.45 (0.85–2.46)	
Race, %						0.09
Mexican American	400	1 [Reference]	0.64 (0.33–1.25)	0.41 (0.18–0.95)	1.28 (0.55–2.98)	
Other Hispanic	295	1 [Reference]	1.58 (0.61–4.08)	1.85 (0.89–3.87)	2.23 (0.69–7.15)	
Non-Hispanic White	832	1 [Reference]	1.12 (0.45–2.80)	2.14 (1.13–4.06)	1.89 (1.13–3.17)	
Non-Hispanic Black	502	1 [Reference]	1.39 (0.61–3.15)	2.07 (0.66–6.50)	2.14 (0.80–5.75)	
Other race	430	1 [Reference]	0.35 (0.10–1.23)	0.38 (0.14–1.00)	0.84 (0.33–2.16)	
Family PIR, %						0.77
≤1.0	699	1 [Reference]	0.93 (0.54–1.60)	0.97 (0.51–1.85)	1.18 (0.60–2.33)	
1.1–3.0	1,012	1 [Reference]	0.79 (0.45–1.39)	1.09 (0.62–1.91)	1.42 (0.78–2.55)	
>3.0	748	1 [Reference]	1.09 (0.52–2.26)	1.52 (0.57–4.05)	2.19 (1.26–3.83)	
Obesity, %						0.72
No	73.6	1 [Reference]	1.07 (0.57–2.00)	1.21 (0.60–2.46)	1.88 (1.06–3.33)	
Yes	26.4	1 [Reference]	0.73 (0.44–1.22)	1.07 (0.67–1.73)	1.20 (0.81–1.78)	

OR, odds ratio; CI, confidence interval. Analyses were adjusted for covariates age (continuous), sex (boys or girls), race (Mexican American, other Hispanic, non-Hispanic White, non-Hispanic Black, or other race), family poverty income ratio (≤1.0, 1.1–3.0, or >3.0), total cholesterol (continuous), high-density lipoprotein cholesterol (continuous), BMI (continuous), hypertension (no, or yes), and diabetes (no, or yes) when they were not the strata variables.

* *P* for interaction were derived using the likelihood ratio test by including interaction terms between the variables of interest in the model.

## 4 Discussion

In this study, we explored the associations between blood manganese and selenium levels and the prevalence of hepatic steatosis among adolescents aged 12–19 based on NHANES 2017–2023 data. We found that higher blood manganese and selenium levels were positively associated with hepatic steatosis prevalence among adolescents. RCS analysis showed linear associations between blood manganese and selenium levels and hepatic steatosis prevalence. Monitoring and maintaining serum manganese and selenium levels at an optimal ranger may be associated with a lower prevalence of hepatic steatosis among adolescents.

Hepatic steatosis is becoming an increasing concern not only in adults but also in younger populations due to rising rates of obesity and metabolic disorders in adolescents. Manganese and selenium are both essential for enzymatic processes and antioxidant defense, but too much or too little can disrupt homeostasis and contribute to disease development. Previous studies have explored the correlations between these trace elements and liver diseases, including hepatic steatosis. Maya et al. conducted an observational study to explore the relationships between five metal exposures with the hallmark features of hepatic steatosis in 2022, including blood cadmium, mercury, lead, manganese, and selenium (31). Among the five metals evaluated, they only found two significant metals. Specifically, serum manganese levels were positively linked to the likelihood of liver steatosis (31). Similarly, higher serum selenium levels were positively correlated with a high risk of liver steatosis, but showed a relationship with a decreased risk of liver fibrosis (31). Recently, Tang et al. also suggested high levels of selenium and manganese were significantly linked with the prevalence of the metabolic dysfunction-associated steatotic liver disease in adults (32). Consistent with previous research, our findings showed that elevated concentrations of selenium and manganese in the blood were positively related with prevalence of hepatic steatosis among adolescents.

A large population-based study used machine learning to analyze the role of 63 specific nutrients on the hepatic steatosis among over 200,000 individuals from UK Biobank and they found dietary manganese intake was inversely related with lower risk of steatosis (10). Chronic exposure to high levels of manganese in occupational settings can lead to liver dysfunction and chronic liver disease mortality (33, 34). While dietary manganese at appropriate levels can provide health benefits, occupational or environmental exposure at high levels leads to toxic outcomes. Multiple studies have reported a positive association between blood manganese level and increased levels of liver enzymes (such as alanine aminotransferase, aspartate aminotransferase, and bilirubin) (9). Animal studies showed that chronic manganese exposure can compromise liver function through oxidative stress and mitochondrial dysfunction, leading to elevated enzyme levels and potentially contributing to chronic liver disease progression (35). Elevated serum manganese concentrations have been linked to an increased risk of all-cause mortality and specifically cardiovascular disease-related mortality in the U.S. population (36). Moreover, maintaining serum manganese levels within the range of 8.67–9.23 µg/L may be beneficial for public health (36).

Chronic manganese exposure may contribute to the development of hepatic steatosis and fibrosis. Manganese is a crucial mineral involved in various metabolic processes. As a cofactor, it plays a significant role in enzymatic reactions. Manganese superoxide dismutase (MnSOD) helps protect cells from oxidative stress by catalyzing the dismutation of superoxide radicals into oxygen and hydrogen peroxide (37, 38). Pyruvate carboxylase is a key manganese-dependent enzyme and is relevant in the context of metabolic disorders (39). Manganese is critical for the activation and proper functioning of pyruvate carboxylase, which catalyzes the conversion of pyruvate to oxaloacetate in gluconeogenesis (39). Manganese is also involved in the synthesis and metabolism of lipids and cholesterol (40). Moreover, manganese levels are essential for supporting gut-liver axis health, potentially reducing the risk of hepatic steatosis (41). By promoting a balanced microbial environment, manganese could indirectly contribute to the prevention of hepatic steatosis (41). For manganese, recommended intake for children varies by age. The Institute of Medicine suggests 1.2–1.5 mg/day for young children and up to 1.9–2.2 mg/day for adolescents, while WHO recommendations range from 0.6 to 3 mg/day (42). However, excessive manganese exposure can be toxic. manganese toxicity can impair enzymes within the electron transport chain, reducing ATP synthesis (43). Manganese exposure can affect mitochondrial membrane permeability and lead to mitochondrial dysfunction and trigger processes such as apoptosis or necrosis (43). To establish clear dose-response relationships between heavy metal exposure and the onset or progression of hepatic steatosis, more longitudinal studies are needed to define the exact concentration thresholds for manganese toxicity.

The relationship between selenium levels and hepatic steatosis appears complex. Several studies have found that elevated selenium levels are associated with an increased risk of hepatic steatosis and hepatic steatosis (44). Previous study suggested that serum selenium concentrations above approximately 130 μg/L were positively associated with both hepatic steatosis and ALT levels (15). Selenium-dependent glutathione peroxidases and other selenoproteins help neutralize reactive oxygen species, thereby reducing oxidative stress and maintaining cellular health (45). At the serum selenoprotein levels of from 70 to 90 μg/L, the antioxidant functions of selenium are fully realized without the potential for toxicity (46). Wu et al. explored the relationship between dietary selenium intake and the prevalence of hepatic steatosis in a middle-aged and elderly population (47). Individuals with higher dietary selenium intake, even less than the recommended selenium intake levels in China, shown a higher likelihood of hepatic steatosis (47).

The recommended daily selenium intake for children varies by age, ranging from 10 to 40 μg/day according to the WHO and the Institute of Medicine (48). These values are lower than the adult recommendation of 55 μg/day due to children's smaller body size and differing needs. Selenomethionine is one of the primary forms of selenium found in dietary sources (49). When consumed in excess, selenomethionine can disrupt normal metabolic processes and potentially lead to adverse effects (50). However, some researchers pointed the potential of selenium as a protective agent in liver health, particularly for individuals at risk of hepatic steatosis and advanced liver fibrosis (16). They also suggested that selenium may not only support liver health but also reduce overall mortality (16). Some researchers demonstrated that selenium exposure can increase liver Protein Tyrosine Phosphatase 1B (PTP1b) activity in rats (51). As PTP1b activity rises, it may hinder the liver's ability to respond to insulin, which is closely linked to the development of hepatic steatosis and other metabolic disorders (51). Research has shown that high dietary selenium intake can lead to elevated plasma triglyceride levels in animal models (52). This suggested that excessive selenium may have adverse effects on lipid metabolism. In summary, while high selenium levels appear to have a potential role in liver fat accumulation based on animal studies, thorough investigations in human populations are essential to validate these findings and clarify the underlying mechanisms.

Adolescence is marked by accelerated cell proliferation, which makes tissues more susceptible to damage from toxins (8). Heavy metals can disrupt DNA replication and interfere with cell cycle regulation, increasing the risk of developmental abnormalities (8). Emerging research pointed that there was a link between high manganese concentrations in the blood and obesity among children and adolescents (53). Environmental toxins like manganese and selenium might contribute to metabolic disorders. While adolescents tend to have lower levels of heavy metal exposure compared to adults, the interactive effects of multiple metals on liver injury and hepatic steatosis may only become apparent at higher levels of exposure. Therefore, more research is needed to assess the impact of trace elements on hepatic steatosis in adolescents.

The study showed the positive associations between trace elements and hepatic steatosis prevalence by analyzing data from 2,459 adolescents. We used vibration-controlled transient elastography for diagnosing hepatic steatosis, which provided a precise and non-invasive assessment of liver fat and could strengthen the validity of the outcomes. Both manganese and selenium showed exhibited linear relationships with hepatic steatosis in adolescents. Further research is needed to better understand the pathways through which manganese and selenium influence liver health.

Our study has several limitations that should be acknowledged. First, the cross-sectional design precludes the ability to establish causality between elevated manganese and selenium levels and the development of hepatic steatosis. Longitudinal studies are warranted to determine whether these trace elements contribute to the onset and progression of hepatic steatosis. Second, while the sample was representative of the U.S. adolescent population, the findings may not be generalizable to other populations or age groups. Further studies in diverse populations are needed to validate our findings and ensure their broader applicability. Third, hepatic steatosis was defined using vibration-controlled transient elastography (VCTE), a widely accepted non-invasive diagnostic tool. However, relying solely on VCTE may not capture the full spectrum of the disease. The diagnosis of hepatic steatosis based exclusively on elastography does not fully align with internationally established guidelines, as recommended by the European Society for Paediatric Gastroenterology Hepatology and Nutrition (ESPGHAN) (54) and North American Society of Pediatric Gastroenterology, Hepatology and Nutrition (NASPGHAN) (55), which advocate for a more comprehensive diagnostic approach incorporating clinical, biochemical, and imaging criteria. The absence of liver biopsy or detailed biochemical assessments, due to the limitations of the NHANES dataset, restricts our ability to fully characterize hepatic steatosis and differentiate it from other metabolic liver diseases. Future studies should consider integrating multiple diagnostic modalities to enhance diagnostic accuracy and adherence to established guidelines. Lastly, although our analysis accounted for common metabolic risk factors, it did not fully address other potential causes of hepatic steatosis. As highlighted in the ESPGHAN position paper (54), factors such as late-onset metabolic diseases (e.g., Wilson disease) and hepatotoxic medications can significantly contribute to hepatic steatosis, especially in pediatric and adolescent populations. These factors may introduce confounding effects that influence liver function and fat accumulation. Future studies should consider incorporating a broader range of metabolic, genetic, and pharmacological factors to provide a more comprehensive understanding of the etiology of hepatic steatosis.

## 5 Conclusion

This study demonstrates a positive association between higher blood manganese and selenium levels and the prevalence of hepatic steatosis in adolescents. Elevated levels of these trace elements were linked to increased liver fat accumulation. Further research is needed to understand the mechanisms behind these associations and to assess their potential implications for public health strategies aimed at preventing hepatic steatosis in adolescents.

## Data Availability

Publicly available datasets were analyzed in this study. This data can be found here: https://www.cdc.gov/nchs/nhanes/.
