# Supplementary material for: Association of blood manganese and selenium levels with hepatic steatosis among adolescents: a nationwide cross-sectional analysis
Source: Front Pediatr. 2025 Feb 11;13:1522219. doi: 10.3389/fped.2025.1522219 (PMC11850372; doi:10.3389/fped.2025.1522219)
Supplement: Supplementary file 1 [file Datasheet1.docx]

**Online Supplementary Material**

**Association of Blood Manganese and Selenium Levels with Hepatic Steatosis Among Adolescents: A Nationwide Cross-Sectional Analysis**

**Table S1.** Survey-weighted characteristics of adolescent participants by quartiles of blood manganese in NHANES 2017–2023.

**Table S2.** Survey-weighted characteristics of adolescent participants by quartiles of blood selenium in NHANES 2017–2023.

**Figure S1.** Spearman correlation analysis among blood manganese and selenium and CAP scores among adolescents in NHANES 2017–2023.

**Table S3.** Multiple linear regression associations of quartiles of blood manganese and selenium levels with CAP scores among adolescents in NHANES 2017–2023.

**Table S1.** Survey-weighted characteristics of adolescent participants by quartiles of blood manganese in NHANES 2017–2023.

| Characteristics | Quartiles of blood manganese levels, μg/L | | | | *P* value |
| --- | --- | --- | --- | --- | --- |
|  | <8.29 | 8.29-10.16 | 10.17-12.61 | >12.61 |  |
| Age, years | 15.68 ± 0.16 | 15.34 ± 0.16 | 15.21 ± 0.09 | 15.25 ± 0.11 | 0.04 |
| Sex, % |  |  |  |  | <0.01 |
| Female | 238 (39.63) | 253 (39.38) | 298 (48.52) | 388 (64.03) |  |
| Male | 377 (60.37) | 364 (60.62) | 314 (51.48) | 227 (35.97) |  |
| Race/ethnicity, % |  |  |  |  | <0.01 |
| Mexican American | 53 (7.62) | 96 (12.81) | 114 (17.32) | 137 (20.87) |  |
| Other Hispanic | 59 (8.95) | 73 (11.97) | 85 (13.19) | 78 (11.55) |  |
| Non-Hispanic White | 223 (52.74) | 217 (50.68) | 215 (48.27) | 177 (39.02) |  |
| Non-Hispanic Black | 207 (20.55) | 137 (13.00) | 91 (8.61) | 67 (7.85) |  |
| Other race | 73 (10.14) | 94 (11.54) | 107 (12.61) | 156 (20.71) |  |
| Family PIR, % |  |  |  |  | 0.62 |
| ≤1.0 | 200 (23.29) | 161 (19.75) | 163 (22.46) | 175 (22.29) |  |
| 1.1–3.0 | 229 (34.80) | 273 (39.98) | 252 (37.72) | 258 (41.01) |  |
| >3.0 | 186 (41.91) | 183 (40.26) | 197 (39.81) | 182 (36.70) |  |
| TC, mg/dL | 153.39 ± 1.58 | 153.73 ± 1.49 | 154.04 ± 1.56 | 158.43 ± 1.47 | 0.02 |
| HDL-C, mg/dL | 52.44 ± 0.51 | 50.65 ± 0.55 | 51.85 ± 0.61 | 52.50 ± 0.65 | 0.01 |
| Body mass index, kg/m^2^ | 23.88 ± 0.40 | 24.16 ± 0.39 | 24.09 ± 0.30 | 25.30 ± 0.28 | 0.002 |
| Hypertension, % | |  |  |  | 0.68 |
| Yes | 568 (94.16) | 577 (93.95) | 578 (95.03) | 584 (95.56) |  |
| No | 47 (5.84) | 40 (6.05) | 34 (4.97) | 31 (4.44) |  |
| Diabetes, % |  |  |  |  | 0.21 |
| No | 615 (100.00) | 609 (99.11) | 605 (99.30) | 609 (99.10) |  |
| Yes | 0 (0.00) | 8 (0.89) | 7 (0.70) | 6 (0.90) |  |
| CAP scores, dB/m | 212.20 ± 2.40 | 219.34 ± 2.53 | 221.66 ± 2.08 | 227.12 ± 3.04 | <0.01 |
| Hepatic steatosis, % | |  |  |  | <0.01 |
| No | 493 (83.21) | 457 (73.12) | 433 (72.11) | 406 (64.50) |  |
| Yes | 122 (16.79) | 160 (26.88) | 179 (27.89) | 209 (35.50) |  |

Abbreviations: PIR, poverty income ratio; TC, total cholesterol; HDL-C, high-density lipoprotein cholesterol; CAP, controlled attenuated parameter. Normally distributed continuous variables are described as means ± SEs, and continuous variables without a normal distribution are presented as medians [interquartile ranges]. Categorical variables are presented as numbers (percentages). N reflects the study sample, whereas percentages reflect the survey-weighted data.

**Table S2.** Survey-weighted characteristics of adolescent participants by quartiles of blood selenium in NHANES 2017–2023.

| Characteristics | Quartiles of blood selenium levels, μg/L | | | | *P* value |
| --- | --- | --- | --- | --- | --- |
|  | <165.30 | 165.30-177.90 | 177.91-192.80 | >192.80 |  |
| Age, years | 15.02 ± 0.16 | 15.11 ± 0.12 | 15.58 ± 0.13 | 15.79 ± 0.13 | <0.01 |
| Sex, % |  |  |  |  | 0.31 |
| Female | 330 (51.46) | 303 (49.39) | 276 (44.40) | 268 (45.79) |  |
| Male | 290 (48.54) | 309 (50.61) | 336 (55.60) | 347 (54.21) |  |
| Race/ethnicity, % |  |  |  |  | 0.19 |
| Mexican American | 80 (12.02) | 104 (14.45) | 115 (16.66) | 101 (15.39) |  |
| Other Hispanic | 104 (17.54) | 64 (9.78) | 66 (9.09) | 61 (9.15) |  |
| Non-Hispanic White | 192 (42.80) | 206 (47.90) | 214 (48.35) | 220 (51.99) |  |
| Non-Hispanic Black | 147 (14.84) | 131 (13.69) | 107 (10.97) | 117 (10.54) |  |
| Other race | 97 (12.80) | 107 (14.18) | 110 (14.93) | 116 (12.93) |  |
| Family PIR, % |  |  |  |  | 0.03 |
| ≤1.0 | 207 (27.41) | 151 (17.57) | 164 (21.50) | 177 (21.15) |  |
| 1.1–3.0 | 234 (34.60) | 273 (40.90) | 253 (40.39) | 252 (37.68) |  |
| >3.0 | 179 (37.99) | 188 (41.54) | 195 (38.11) | 186 (41.17) |  |
| TC, mg/dL | 150.56 ± 1.38 | 152.21 ± 1.32 | 156.65 ± 1.90 | 160.22 ± 1.58 | <0.01 |
| HDL-C, mg/dL | 52.75 ± 0.69 | 51.80 ± 0.54 | 51.80 ± 0.79 | 51.02 ± 0.79 | 0.48 |
| Body mass index, kg/m^2^ | 24.49 ± 0.48 | 23.92 ± 0.30 | 24.40 ± 0.35 | 24.60 ± 0.38 | 0.39 |
| Hypertension, % | |  |  |  | 0.01 |
| Yes | 588 (95.49) | 593 (97.80) | 566 (92.96) | 560 (92.39) |  |
| No | 32 (4.51) | 19 (2.20) | 46 (7.04) | 55 (7.61) |  |
| Diabetes, % |  |  |  |  | 0.60 |
| No | 615 (99.32) | 608 (99.43) | 609 (99.69) | 606 (99.06) |  |
| Yes | 5 (0.68) | 4 (0.57) | 3 (0.31) | 9 (0.94) |  |
| CAP scores, dB/m | 218.80 ± 2.09 | 215.46 ± 1.81 | 220.74 ± 3.58 | 225.30 ± 3.61 | 0.10 |
| Hepatic steatosis, % | |  |  |  | 0.03 |
| No | 459 (74.76) | 477 (77.72) | 443 (72.65) | 410 (67.82) |  |
| Yes | 161 (25.24) | 135 (22.28) | 169 (27.35) | 205 (32.18) |  |

Abbreviations: PIR, poverty income ratio; TC, total cholesterol; HDL-C, high-density lipoprotein cholesterol; CAP, controlled attenuated parameter. Normally distributed continuous variables are described as means ± SEs, and continuous variables without a normal distribution are presented as medians [interquartile ranges]. Categorical variables are presented as numbers (percentages). N reflects the study sample, whereas percentages reflect the survey-weighted data.

**
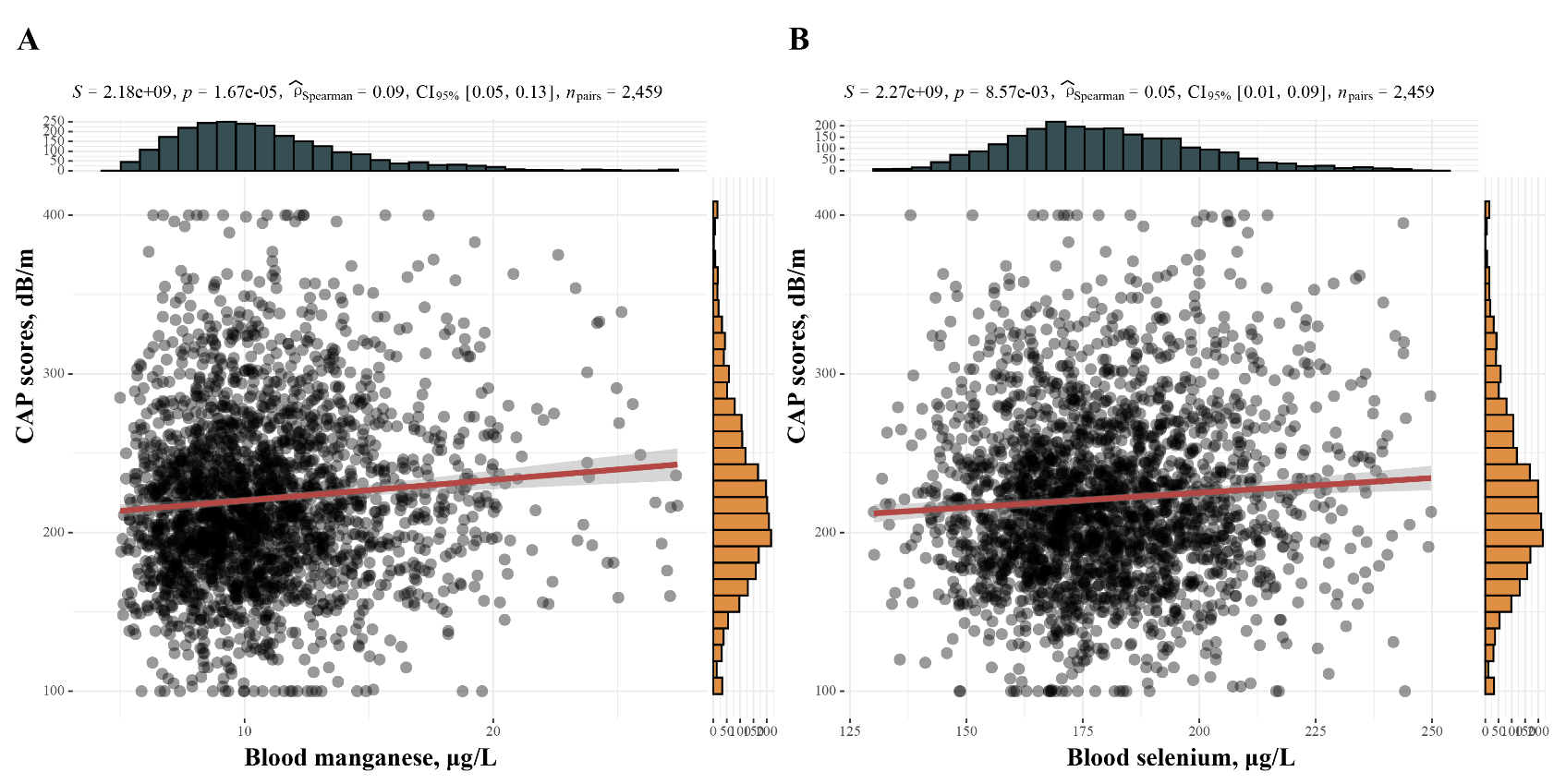
**

**Figure S1.** Spearman correlation analysis among blood manganese and selenium and CAP scores among adolescents in NHANES 2017–2023.

**Table S3.** Multiple linear regression associations of quartiles of blood manganese and selenium levels with CAP scores among adolescents in NHANES 2017–2023.

|  | Quartiles of blood manganese and selenium levels | | | |  |
| --- | --- | --- | --- | --- | --- |
|  | β | β (95% CI) | β (95% CI) | β (95% CI) | *P* _trend_ |
| Blood manganese | |  |  |  |  |
| Crude | 0 [Reference] | 7.13 (0.35, 13.91) | 9.45 (3.79, 15.12) | 14.91 (6.17, 23.66) | <0.01 |
| Model 1 | 0 [Reference] | 6.82 (-0.23, 13.87) | 9.26 (3.51, 15.01) | 15.03 (7.57, 22.49) | <0.01 |
| Model 2 | 0 [Reference] | 3.38 (-1.90, 8.66) | 6.25 (1.80, 10.71) | 6.75 (0.14, 13.36) | 0.02 |
| Blood selenium | | |  |  |  |
| Crude | 0 [Reference] | -3.34 (-9.79, 3.10) | 1.94 (-7.35, 11.22) | 6.5 (-0.37, 13.36) | 0.02 |
| Model 1 | 0 [Reference] | -3.33 (-9.53, 2.86) | 0.7 ( -7.86, 9.25) | 5.54 (-0.95, 12.04) | 0.04 |
| Model 2 | 0 [Reference] | -0.77 (-6.70, 5.15) | 1.38 (-5.98, 8.74) | 5.47 (0.70, 10.23) | 0.02 |

Abbreviations: CI, confidence interval; CAP, controlled attenuated parameter.

Model 1 was adjusted for age (continuous), sex (boys or girls), and race (Mexican American, other Hispanic, non-Hispanic White, non-Hispanic Black, or other race); Model 2 was adjusted for Model 1 plus family poverty income ratio (≤1.0, 1.1–3.0, or >3.0), total cholesterol (continuous), high-density lipoprotein cholesterol (continuous), BMI (continuous), hypertension (no, or yes), and diabetes (no, or yes).
